# Sharing of gut microbial strains between selected individual sets of twins cohabitating for decades

**DOI:** 10.1371/journal.pone.0226111

**Published:** 2019-12-05

**Authors:** Hyunmin Koo, Joseph A. Hakim, David K. Crossman, Elliot J. Lefkowitz, Casey D. Morrow

**Affiliations:** 1 Department of Genetics and Heflin Center for Genomic Science, University of Alabama at Birmingham, Birmingham, Alabama, United States of America; 2 Department of Biology, University of Alabama at Birmingham, Birmingham, Alabama, United States of America; 3 Department of Microbiology, University of Alabama at Birmingham, Birmingham, Alabama, United States of America; 4 Department of Cell, Developmental and Integrative Biology, University of Alabama at Birmingham, Birmingham, Alabama, United States of America; University of Illinois at Urbana-Champaign, UNITED STATES

## Abstract

**Background:**

Given the increasing realization of the important functions of the gut microbial community in human health, it is important to determine whether the increased age of the host coupled with inevitable environmental changes can alter the stability of individual microbial strains of the gut microbial community. Since early studies demonstrated that pairs of twins possess the related gut microbial communities, to gain insights into the temporal stability of the reservoir of gut microbial strains in humans, we have assessed the strain relatedness of samples from two previously published data sets that were obtained from twin children and adults (36–80 years old) who have been either living together or apart for different times.

**Methods:**

We analyzed the two data sets; twin children (*n* = 24) and adults (*n* = 50) using our previously developed strain-tracking program called Window-based Single Nucleotide Variant (SNV) Similarity (WSS) that can distinguish a related strain pair from a non-related strain pair based on the overall genome-wide SNV similarity. To independently substantiate the identification of distinct microbial genomic variants (herein strains) observed from WSS analysis, we used analysis by StrainPhlAn.

**Results:**

Analysis of the twin children data set revealed a significantly (*P*-value <0.05) higher number of the shared strain pairs with a predominance of *Bacteroides vulgatus* between individual sets of twin pairs than the twin adult data set. Additional analysis on the adult twins showed that twins who have been living apart less than 10 years shared significantly more related strain pairs than twins living apart between 10 to 60 years. Eighty-year-old twins who had been living together for 79 years then separated for 1 year showed the highest number of related strain pairs consisting of *B*. *vulgatus*, *Eubacterium eligens*, and *Bifidobacterium adolescentis*. The next highest number of related strain pairs was found in 56-year-old twins who had been living together for 51 years then separated for 5 years (*B*. *vulgatus* and *Coprococcus eutactus* as related strains), 73-year-old twins living together for 66 years and then separated for 7 years (*Bacteroides uniformis and Clostrium sp*. *L2-50* as related strains) and 36-year-old twins separated for 19 years (shared strains of *Alistipes shahii* and *E*. *eligens*). Finally, a sporadic appearance of a single shared strain that did not show a correlation with time of separation was observed in three twin sets that had separation times between 22 to 54 years.

**Conclusion:**

We conclude from our strain-tracking analysis of twins that certain gut microbial strains can be shared between individuals in some cases for decades. Changes in the host environmental conditions over time can impact the stability landscape of the gut microbial community resulting in the appearance of new strains that could potentially impact microbe interactions that are essential for function in human health.

## Introduction

There has been a growing interest into the origin and development of the human gut microbial community. Recently, the inheritance of certain gut microbes, such as those from Actinobacteria and Bacteroidia, has been shown to occur from the mother to the infant indicating the infant is probably seeded with multiple strains creating a microbial reservoir in the gastrointestinal tract [1,2]. During the first three years or so after birth, the infant gut microbiome undergoes a series of short-term changes in microbial community composition that resolves into a dominant strain creating a stable community that is resistant to disruption [3–8].

An unresolved issue is the long-term temporal stability of the microbes (and their progeny). Alteration of the gut microbial community structure as a result of aging and environmental changes such as diet or antibiotics could impact the stability of individual gut microbial strains resulting in changes in the composition of the gut microbial community. Given the role of the gut microbial community in host metabolism, protection against invading pathogens and immune function, these alterations in community structure could have profound consequences for the health of the host [9–13].

In order to investigate the intricacies of the gut microbial community structure, it is necessary to use shotgun metagenomic sequencing that allows for the identification of microbial genomic variants (herein strains) [14–16]. In a previous study, we developed a unique strain-tracking program called Window-based Single Nucleotide Variant (SNV) Similarity (WSS) to assess the strain relatedness of multiple microbes in two separate samples [14]. In this program, the cut-off value for each strain’s relatedness was established based on the Human Microbiome Project (HMP) data set and used to differentiate a related strain pair (both strains were taken from the same individual at separate times) from a non-related strain pair (both strains were taken from different individuals). The finding from the WSS analysis on the HMP data set showed that microbial strains are unique between individuals, which can also support the concept of microbiome fingerprint [14,17,18]. We have also used the WSS analysis to track related strains in individuals following antibiotic(s) treatment or a fecal transplant for recurrent *C*. *difficile* or gastric bypass surgery that disrupts the physiological environment of the small intestine[14,19,20].

Previous studies have demonstrated that individual pairs of twins have similar gut microbiome compositions, consistent with the idea that the microbes were inherited from the mother and the twins would have very similar environmental exposure to other microbes [21,22]. Moreover, the long-term stability (over 5 years) of the gut microbial strains in individuals and between family members including mothers and their twin pairs have reported using 16S rRNA targeted sequencing approaches [23]. In the current study, we have re-analyzed data sets of twin children and adults at ages ranging up to 80 years old (with up to 59 years of separation) to evaluate the sharing of the gut microbial strains using the WSS followed by StrainPhlAn analyses. Our results on the sharing of certain microbial strains from child and adult twins show the potential for the long-term stability of certain gut microbes in the human gastrointestinal tract.

## Materials and methods

### Twin data sets

We used two publicly available data sets, 1) Korpela et al. [1] and 2) Xie et al. [21], to conduct strain-tracking analysis. For Korpela et al., 4 available children twin sets including 1 from the Netherlands and 3 from Germany were downloaded and used for the analyses. For each individual, 3 fecal samples were collected at different time points (Days 0, 7, and 28 for the Dutch family, and Days 0, 7, and 30 for the German family). In total, 24 samples (3 samples for each individual) were downloaded and used in this study to perform strain-tracking analysis. Due to a low sequence number and coverage to run WSS analysis, 1 sample was excluded from the 24 samples (**S1 Table**). To compare with the twin children data set, we have downloaded the selected 25 adult twin sets out of 125 sets from Xie et al. based on the days that twins have been living apart (1–9, 10–19, 20–29, 30–39, 40–49, and 50–59 years). A subset of 25 adult twin sets was conducted due to a limited number of samples (P47, P7, and P54) that were available to represent the 1–9 years separation time point [20]. In total, 50 samples were used in this study to perform strain-tracking analyses (**S1 Table**).

### Total sequence reads and processing

A total of 2,462,958,975 metagenomics sequencing reads were downloaded from the two data sets; 667,212,374 reads from the Korpela et al. and 1,795,746,601 from the Xie et al. (**S1 Table**). Sequence reads were then filtered to remove adapters, low quality reads (sliding window of 50 bases having a QScore <20), and short sequences (sequence length <50 bases) using Trimmomatic (version 0.36)[24]. After quality-based trimming and filtering processes, a total 2,381,182,671 sequences were used for the downstream analyses (**S1 Table).**

### Strain-tracking analyses

From twin children and adults data sets, each twin set (*i*.*e*. Twin 1 and Twin 2 from the same biological parents) was separately used to conduct the strain-tracking analyses.

For the WSS analysis, high-quality processed reads were aligned to the 93 reference sequences, which were common and dominant in stool samples collected from healthy European and North American [14,17] using the Burrows-Wheeler aligner tool BWA-MEM (version 0.7.13) with the “-M” option [25]. Mapped reads were then filtered to exclude reads that mapped to multiple locations or resulted in a low percent match (<90%) using “mgSNP_sam-filter.py” implemented in the WSS. Before calling variants, the filtered reads were sorted (“SORT_ORDER = coordinate”), marked for duplicates (“MarkDuplicates,VALIDATION_STRINGENCY = SILENT,CREATE_INDEX = True, ASSUME_SORTED = True”) using Picard Toolkit (version 1.129, http://broadinstitute.github.io/picard/), and then used for indel realignment (“-T RealignerTargetCreator” for generating targets for indel realignment and “-T IndelRealigner, -targetIntervals” for indel realignment) using Genome Analysis Toolkit (GATK; version 3.7)[26]. SNVs were called for each reference sequence in all samples using GATK with the “-T HaplotypeCaller,—sample_ploidy 1—emitRefConfidence BP_RESOLUTION” options. Multi-sample SNVs for each given reference sequence were measured among all samples for each twin set using GATK with the “-T GenotypeGVCFs—sample_ploidy 1—variant—includeNonVariantSites” options. Using the WSS workflow, the resultant multi-sample Variant Call Format (VCF) files were then used to extract the SNV information for every possible pair of samples for each microbial species using the “run_mgSNP_cov.sh” code. The resultant annotated VCF file was then used for pairwise comparison of the genomic windows determined for each strain [14] using the “mgSNP_compare.sh” code, as implemented in the WSS. The WSS score (%) was calculated by identifying the genomic windows which have no SNV pattern difference between the pair of samples out of the total “good/usable” windows [14]. Any sample having low sequence coverage (<30%) and low sequence depth (<3.5) against their given reference sequences were excluded from the pairwise comparisons (**S2 Table**). In addition, low coverage windows with more than 50% of the bases having a read depth <5 were ignored when comparing the SNV similarity between sample pairs. All codes implemented in the WSS were deposited by our previous study [14] and are available at https://github.com/ranjit58/mgSNP.

From the WSS analysis, a total of 20 and 25 species were detected from the Korpela et al. and Xie et al. data sets, respectively. To distinguish a related strain pair for each twin set (*i*.*e*. related strain pair between Twin 1 and Twin 2 from the same biological parent), a WSS score for each species was compared against each species’ cut-off value that was established based on the HMP data set in our previous study (Related strain pair: WSS score > cut-off; Unrelated strain pair: WSS score < cut-off) [14,19]. The resultant comparison analysis for all twin sets was summarized and visualized using Microsoft Excel (Microsoft, Seattle, WA, USA). To predict the chance of misclassifying unrelated samples as related, all individual samples from the children twin sets and a subset of 8 adult twin sets (P47, P7, P54, P53, P5, P82, P29, and P97) were separately used to run WSS analysis.

Strain-tracking analysis for *Bacteroides vulgatus* was additionally conducted on the twin adult data set using StrainPhlAn using default parameters and with the options “—relaxed_parameter3,—marker_in_clade 0.1” [16]. To do this, the high-quality processed reads were mapped against the set of species-specific marker gene database established in MetaPhlAn [27,28]. The sample-specific markers were reconstructed by using the variant calling approach, and then the reconstructed markers were used to build a phylogenetic tree of the strains [16]. The resultant phylogenetic tree for *B*. *vulgatus* was visualized using the neighbor-joining method in Jalview using default parameters [29].

### Statistical analysis

Statistical significance (P-value <0.05) was determined by using one-way ANOVA followed by Tukey’s multiple-comparisons *post hoc* test in R (version 3.5.1), as appropriate and indicated in the main text and figure legends [20]. Elaborated values were shown in **S3 Table**.

## Results

The vertical transmission and persistence for at least 1 year of certain gut microbial strains from the class Actinobacteria and Bacteroidia from mothers to infants have previously been described by Korpela et al. [1]. Since twins would have a common mother and probably similar environmental conditions throughout childhood, we would anticipate they might share certain gut microbial strains [21]. To substantiate this possibility, we used WSS analysis followed by StrainPhlAn to determine whether any strains were shared between the individual children twin pairs data set in Korpela et al. [1]. We also used a unique data set that comprised adult twins between 36–80 years old that have been living either together or apart for different time periods [21] (**S1 Table**). For further comparison analysis, we sub-grouped the adult twin data set based on decades post-separation.

**Fig 1:**
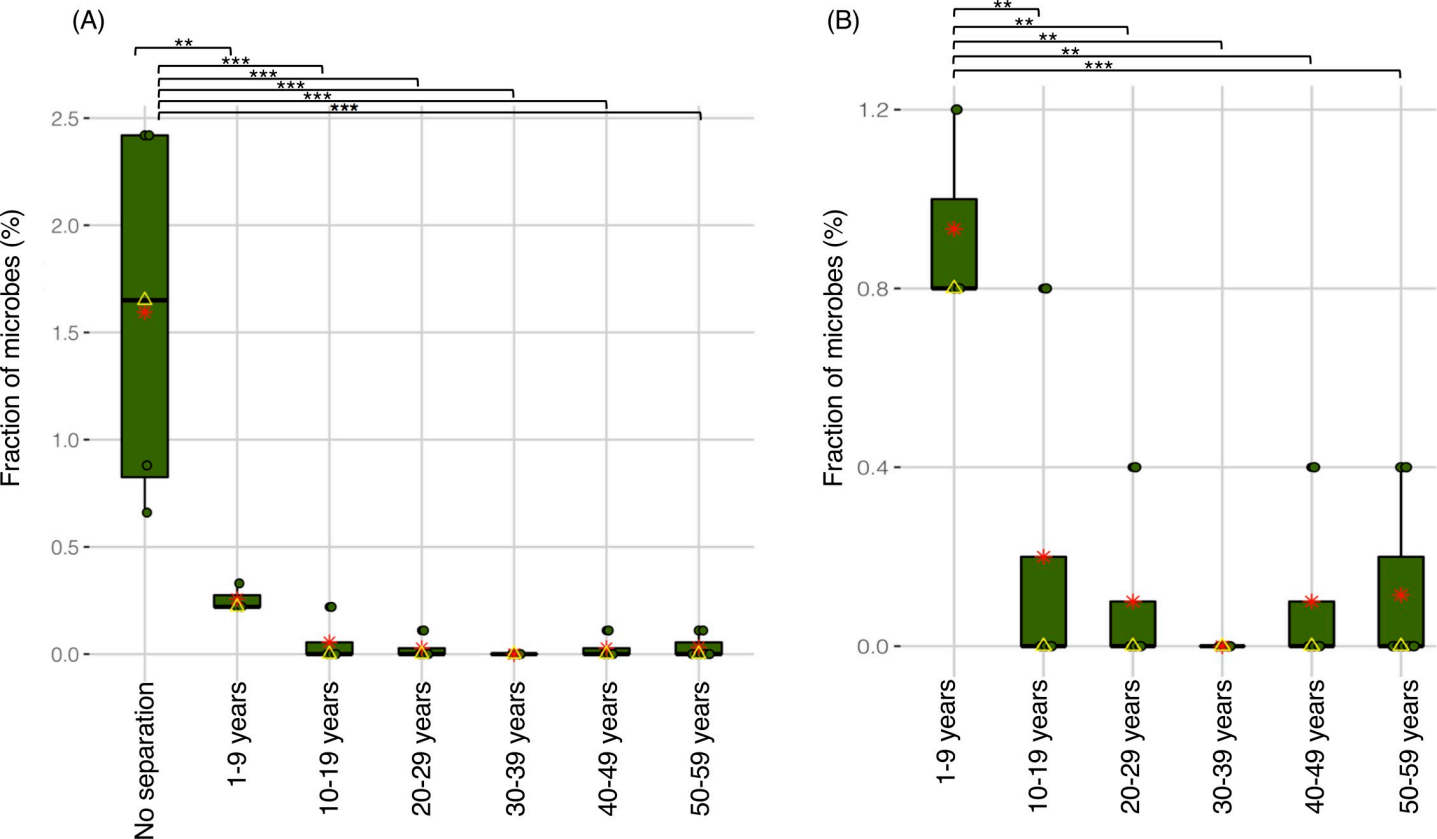
Related strains observed across all twin pairs over separation time points. Boxplots represent a fraction of the related strain found in each twin pair obtained from two data sets (twin children data from Korpela et al., and twin adult data from Xie et al.) at different separation time point intervals (no separation, 1–9, 10–19, 20–29, 30–39, 40–49, and 50–59 years). All twin children data were clustered into the no separation time point, and all twin adults data were sorted into 6 different separation time point intervals based on the days that twins have been living apart. The boxplots display the median (a yellow triangle), mean (a red asterisk), and the interquartile range. Each dot in the boxplot shows a value observed per each twin pair, and the whiskers of the box are extended to the lowest and highest value found in each separation time points. Boxplots were generated using the ggplot2 package (version 3.1.1) in R (version 3.5.1). **(A)** Significant differences (*P*-value <0.05) between no separation vs. each separation time point interval were tested using an ANOVA followed by Tukey’s multiple-comparisons *post hoc* tests in R (version 3.5.1), and represented as a black asterisk above the boxplot; *P-value <0.05, **P-value <0.01, ***P-value <0.001, n.s. = not significant (See **S3 Table** for detailed values). **(B)** Significant differences (*P*-value <0.05) within the various separation time point intervals, specifically when 1–9 years was compared to the rest of the time point intervals 10–19, 20–29, 30–39, 40–49, and 50–59 years) were tested using the same statistical analysis in the panel (A).

The fraction of microbes (%) represented a proportion of the related strains (WSS scores > cut-off values) in relation to the total number of strains observed in both data sets (calculation for the fraction of microbes was elaborated in **S3 Table** legend). The data set from twin children showed a significantly increased fraction of the related strains between each twin pair as compared to the twin adult data set, demonstrating the increased sharing of microbial strains between the sets of twin children compared to that of adult twins (**Fig 1A and S3 Table).** We further analyzed the sharing of strains within the twin adult data set and noted that the twins with the least time of separation (less than 10 years) significantly sharing more strains than the groups with longer times of separation (**Fig 1B**).

### Identification of shared microbial strains in twins

**Fig 2:**
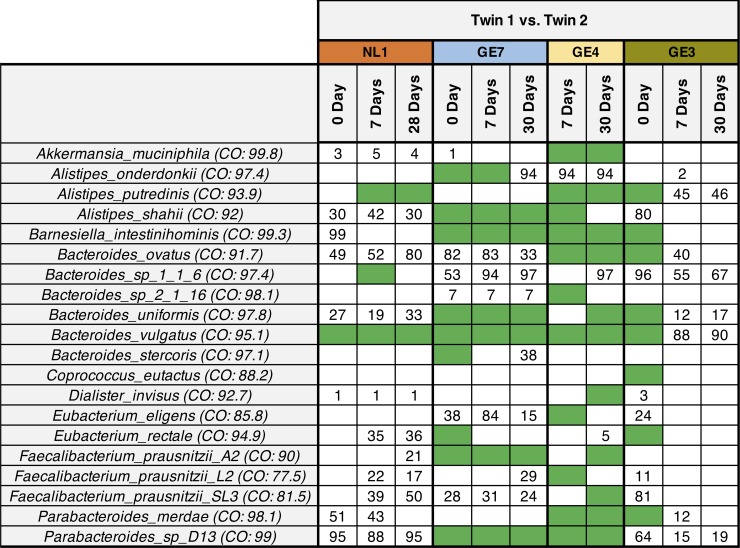
Summarized WSS scores for children twin sets. A total 20 species that were abundant across all children twin pairs obtained from Korpela et al. were selected to compare the WSS scores between each twin set (*i*.*e*. Twin 1 vs. Twin 2) from the same family over ~1 month; 28 days for Dutch family (NL1), and 30 days for German family (GE3, GE4, GE7). Each column in the table shows an individual twin set at different time points and clustered into the same family (4 different colors above the time points). Detailed sample information is shown in the **S1 Table**. The subsequent WSS relationships between each species were based on the cut-off values previously established by Kumar et al. to distinguish a related strain pair (WSS score > cut-off) from a non-related strain pair (WSS score < cut-off). The green boxes show that the related strain pair were observed between Twin 1 and Twin 2 from the same biological parents at a certain time point. The white boxes represent the microbial strains that we were unable to reliably determine relatedness due to the majority of the sample pairs not satisfying the criteria of WSS analysis (both samples are required to have minimum coverage > 30% and average depth > 3.5), or abundances of the species was low/absent (taxonomic composition data was reported in Korpela et al. and Xie et al.). The numbers shown in the white boxes represent the WSS scores (%) which were obtained from the pairwise comparison between Twin 1 and Twin 2 (the higher WSS scores indicate more similar/identical windows were identified between Twin 1 and Twin 2).

Considerable strain sharing was found for the most of children twin sets (3 out of 4) for several microbes, notably *B*. *vulgatus* and *Alistipes putredinis* (**Fig 2**). We found that the Netherland twin set (NL1) shared *B*. *vulgatus* strain for all three of the time points, and shared *A*. *putredinis* for the two-time points (**Fig 2**). We also found a considerable number of strains were shared between the German children twin sets (GE4 and GE7) with a higher number of microbes included members of genera *Akkermansia*, *Alistipes*, *Barnesiella*, *Bacteroides*, *Eubacterium*, *Faecalibacterium*, and *Parabacteroides* as compared to the NL1. Not all twin pairs though had shared of microbial strains; we found limited sharing of the Day 0 sample for the twin set of GE3 family consisting of members of genera *Alistipes*, *Barnesiella*, *Bacteroides*, *Coprococcus*, *Eubacterium*, *and Parabacteroides*.

**Fig 3:**
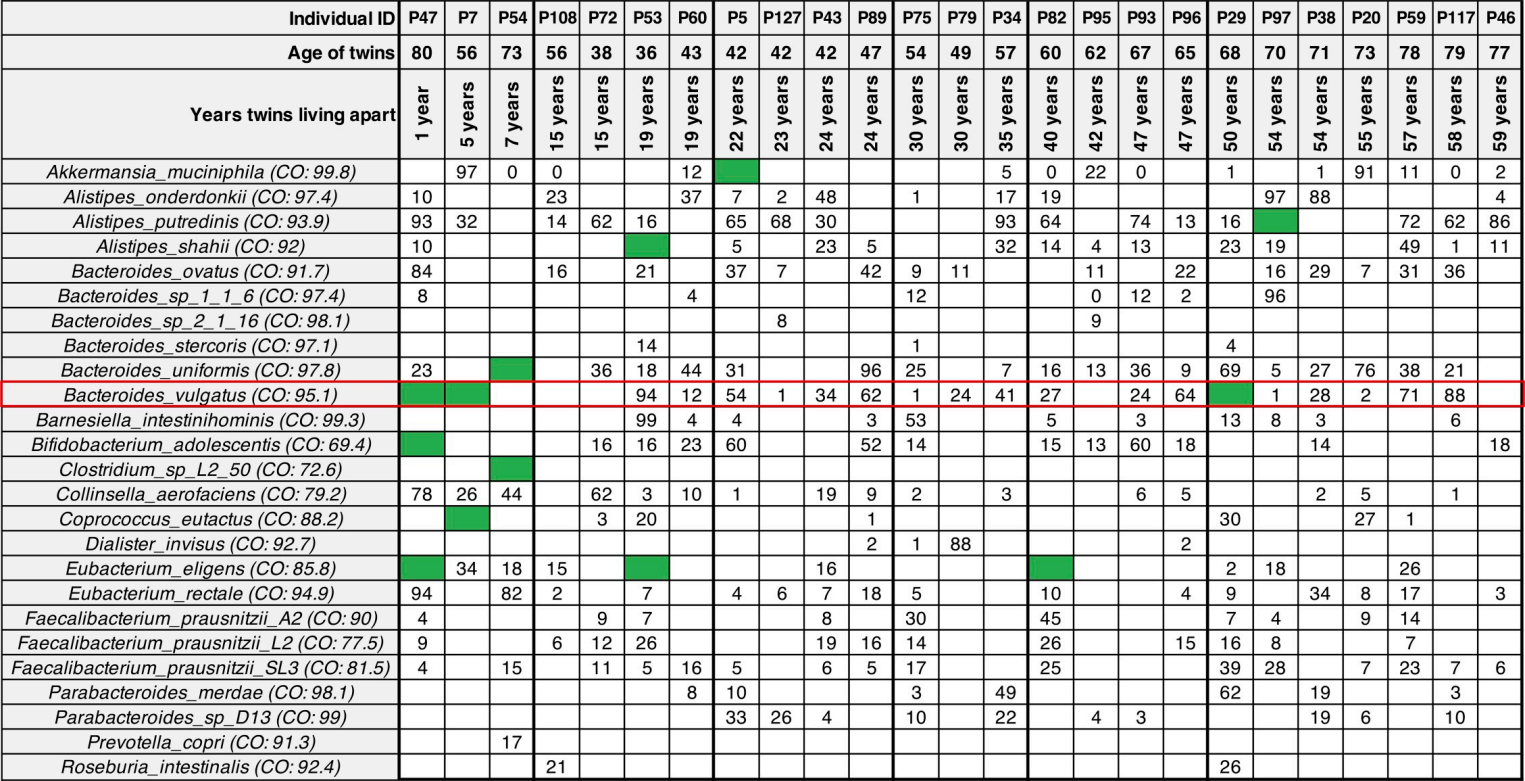
Summarized WSS scores for adult twin sets. A total 25 species that were abundant across all adult twin pairs obtained from Xie et al. were selected to compare the WSS scores between each twin set (*i*.*e*. Twin 1 vs. Twin 2) at various separation time points (from 1 to 59 years; time point intervals were represented by the thick vertical line in the table). Each column in the table represents an individual twin set that had a different age, separation time point and matches to the sample information listed in the **S1 Table**. The subsequent WSS relationships between each species were determined based on the cut-off values (See Fig 2 legend). The green boxes represent strain pairs that were related between Twin 1 and Twin 2, from the same biological parents. The white boxes show microbial strains that we were unable to reliably determine relatedness due to the majority of the sample pairs not satisfying the criteria of WSS analysis (both sample are required to have minimum coverage > 30% and average depth > 3.5), or abundances of the species was low/absent for the majority of the sample pairs. The numbers shown in the white boxes represent the WSS scores (%) which were obtained from the pairwise comparison between Twin 1 and Twin 2 (the higher WSS scores indicate more similar/identical windows were identified between Twin 1 and Twin 2). The additional strain-tracking analysis was conducted for *B*. *vulgatus* from the individual twin set P47, P7, and P29 (Red outlined boxes; result from this analysis shown in **Fig 4**).

The adult twin pairs were 36–80 years old that have been living together or apart for different times [21]. To determine whether individual sets of twin pairs shared specific microbial strains, we analyzed a subset of 25 twin pairs that were selected to include those with the shortest (1 year) and longest (79 years) separation times using WSS analysis. From the one 80-year-old twin pair (P47) who had been living together for 79 years and separated for 1 year, we identified the highest number of shared strains consisting of *B*. *vulgatus*, *Eubacterium eligens*, and *Bifidobacterium adolescentis* across all adult twin sets (**Fig 3**). For the one 56-year-old twin pair (P7) who had been living together for 51 years then separated for 5 years, 2 species (*B*. *vulgatus* and *Coprococcus eutactus*) were found to be shared strains (**Fig 3**). Similarly, 73-year-old twins (P54) living together for 66 years and then separated for 7 years showed 2 shared strains of *Bacteroides uniformis*, *and Clostridium sp*. *L2-50* between the twin pair (**Fig 3**).

We noted that in the remaining 22 adult twin sets that had more than 10 years of separation times, there was a sporadic appearance of shared strains that did not show a correlation with time of separation: 5 strains within species of *Akkermansia muciniphilia*, *A*. *putredinis*, *Aliistipes shahii*, *B*. *vulgatus*, and *E*. *eligens* were related between certain twin set (**Fig 3**). In one of these strains, *E*. *eligens*, three sets of twins included P47 (1 year apart, 80-year-old), P53 (19 years apart, 36-year-old), and P82 (40 years apart, 60-year-old) showed a related strain pair. To further investigate these shared strains, we determined the chance of misclassification by analyzing the WSS for all sample pairs from the 8 subsets of adult twins that had related strains (P47, P7, P54, P53, P5, P60, P68, and P70). For the adult twin subsets, the rate of misclassification of *E*. *eligens* was 0.12% (*i*.*e*. 2 unrelated individual pairs showed the WSS scores above the cut-off values out of 1544 pairs) (Values for other microbes are listed in the **S4 Table.** In addition, analysis of the twin children is also present in the **S4 Table**). However, these rates were considerably low as compared to our previous findings on the HMP data set, which was 7% [14,19].

**Fig 4:**
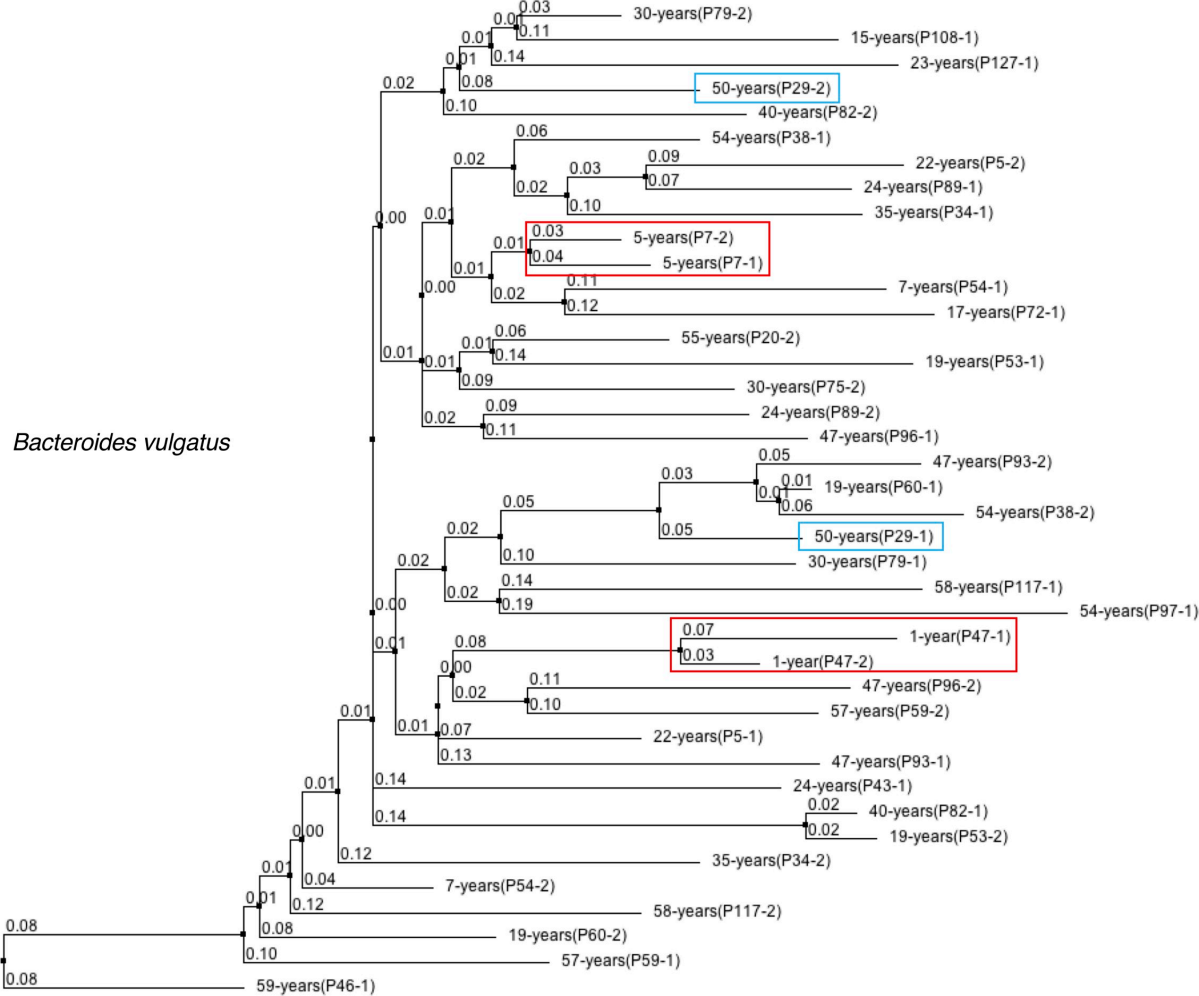
Phylogenetic tree at strain-level using StrainPhlAn. DNA sequences from species-specific marker genes were aligned to all adult twin sets used in this study. Then, a neighbor-joining phylogenetic tree was built based on percentage identity (PID) distance between the marker genes through Jalview (see Methods). The numbers at joining nodes indicate a PID. Here we show one bacterial species, namely *Bacteroides vulgatus*, confirming the presence of the related strain in the two adult twin pairs (red outlined boxes) based on both WSS and StrainPhlAn. We also found the presence of two unrelated strains in one adult twin pair based on StrainPhlAn that were determined as related through WSS analysis (blue outlined boxes).

Finally, to further validate the WSS analysis, we have used StrainPhlAn, which uses a different algorithm based on a species-specific marker gene database to assess strain relatedness for all twin pairs that had shared strains [16]. In the analysis of the *B*. *vulgatus*, we found only one case where the WSS and StrainPhlAn were in disagreement (**Fig 4**). Although two twin pairs (P47 and P7) who had a separation time less than 10 years showed a related *B*. *vulgatus* strain between each twin pair using both WSS and StrainPhlAn, one twin pair (P29) who had separated for 50 years showed an unrelated *B*. *vulgatus* strain when StrainPhlAn was applied (**Fig 4**). All other shared microbial strains identified using WSS were in agreement when analyzed by StrainPhlAn.

## Discussion

The new insight from the current study was the demonstration of the long-term longitudinal stability of certain microbial strains in humans. In previous studies, we, and others have shown the stability of the microbial strains in the gut microbial community with an individual between adults of 1–2 years and infants for up to 5 years [14,17,19,23]. Our approach took advantage of recent studies that have demonstrated the transmission of certain microbial strains from mother to infants and, as shown by our analysis of the 4 twin children data set, revealed a considerable number of shared microbes between each of the young twin pairs probably at an age where they cohabitated the same household [1]. We also analyzed a unique data set with samples from adult twins at ages from 36 to 80 years old [21]. Here we found that samples taken from 3 sets of twins with the least time of separation (1–7 years) had multiple shared microbial strains; twins separated for 19, 22, 40, and 54 years each showed a shared a microbial strain (**Fig 3**). Collectively, these results support that microbial strains possibly remain in certain individuals for longer times than previously identified, possibly up to a decade [14,17,19,23].

We acknowledge there are potential limitations to this interpretation. Only a single time point was available for the adult twins and it is not clear that those microbes have been maintained over the life of the individuals (*e*.*g* up to 80 years in one set of twins), or whether they were recently acquired shared strains between pairs of twins. Since we do not have extensive metadata information throughout the twin’s lifetime, it is possible that one of the twins had known disruptors of the gut microbial community, such as antibiotics, that might have compromised the strains in the microbial community. The same strains that could have been shared by the other co-habiting twin following the recovery of the microbial community after antibiotics [20,30,31]. We also cannot discount that one of the twins might have acquired the new strain from an outside source that was then also shared with the other twin, although this would seem unlikely after the twins had separated. Additional studies with more longitudinal samples would be needed to resolve these possibilities.

Finally, a recent study by Garud et al. modeled the dynamics of the evolution of the human gut microbial community [32]. Their analysis, which was based on fecal samples, described the evolution that consisted of a compilation of single nucleotide changes resulting from replication. The source of the strains found in the fecal samples used for the analysis is most probably the reservoir in the niches that the microbes inhabit in the gastrointestinal tract [9,33]. The long-term sharing of the certain microbial strains as determined by the separation times for the adult twins suggests the possibility for a slower single nucleotide change in the individual’s gastrointestinal tract niches. It is possible that those microbes with the low nucleotide changes for long-term are involved in a form of persistence as an evolutionary strategy to tolerate extended disruptions such as dietary shifts, infection, or antibiotic treatment. A form of persistence may also allow beneficial microbes to recover following perturbations that could potentially restore the stability landscape of the microbial community resulting in a composition similar to that prior to the disruption [3,9–13,24,34].

## Conclusion

The WSS strain-tracking analyses of the child and adult twins demonstrated the existence of individual-specific and shared microbial strain(s) between pairs of twins. Moreover, the adult twins ages from 36 to 80 years old showed a certain strain(s) between pair of twins was shared post-separation. We conclude that the potential exists for an individual to maintain the same strains of certain gut microbes for an extended time, possibly decades. While we do not know the origin of these shared microbes in the twins, these results suggest the possibility of strains shared between non-cohabitating twins for decades. However, for most individuals, environmental changes such as antibiotics and new diets that occur during aging can disrupt the stable landscape of the gut microbial community leading to the emergence of new strains. Numerous studies have described the importance of the gut microbial community interactions needed for essential functions in metabolism, training of the immune system and prevention of colonization by pathogens which could be impacted by these strain changes [3,9–13].

## Supporting information

S1 Table Sequence reads information of each sample used in this study. The original sequence files were sequenced, preprocessed, and deposited by (A) Korpela et al. and (B) Xie et al. To conduct the WSS analysis, all files were downloaded from the European Nucleotide Archive (accession numbers: PRJEB24041 and ERP010708). The table represents the sequence read count before and after filtering processes.
(XLSX)

S2 Table Sequence coverage (%) and depth. Samples from (A) children twin sets (Korpela et al.) and (B) adult twin sets (Xie et al.) were used to check DNA sequence coverage and depth against each given reference sequence. Any sample that had low sequence coverage (<30%) and low sequence depth (<3.5) against their given reference sequences was not listed and excluded for the analysis. (C) The average of sequence coverage and depth was calculated for children and adult twin sets, separately.
(XLSX)

S3 Table Statistical tests. Significant differences (P-value < 0.05) of a fraction of the related strains between (A) two data sets (Children twins vs. Adult twins) and within (B) Adult twins data set (Children twins data set from Korpela et al. and Adult twins data set from Xie et al.). For comparison between children and adult twin sets, the fraction of microbes (%) per each twin pair was calculated by (the number of related strains) x 0.0011. (0.0011 was obtained by 1 divided by the total number of strains in two data sets (*n* = 845)). For comparison within adult twin sets, the fraction of microbes (%) per each twin pair was calculated by (the number of related strains) x 0.004. (0.004 was obtained by 1 divided by a total number of strains in adult data sets (*n* = 250)). Analyses were conducted using an ANOVA followed by Tukey's multiple-comparisons post hoc tests in R (version 3.5.1). The *P*-value was adjusted by using the TukeyHSD function in R (version 3.5.1).
(XLSX)

S4 Table WSS analysis. All individual samples from (A) children twin sets (Korpela et al.) and (B) a subset of 8 adult twin sets (P47, P7, P54, P53, P5, P82, P29, and P97) from Xie et al. were used to run WSS analysis. Related strains (WSS score > cut-off value) between sample 1 and sample 2 were colored in red.
(XLSX)

## Abbreviations

WSS: Window-based Single Nucleotide Variant Similarity
HMP: Human Microbiome Project
SNV: Single Nucleotide Variant
